# Immunolocalization of Some Epidermal Proteins and Glycoproteins in the Growing Skin of the Australian Lungfish (*Neoceratodus forsteri*)

**DOI:** 10.3390/jdb11030035

**Published:** 2023-08-14

**Authors:** Lorenzo Alibardi

**Affiliations:** 1 Comparative Histolab Padova, 35100 Padova, Italy; lorenzo.alibardi@unibo.it; 2 Department of Biology, University of Bologna, 40126 Bologna, Italy

**Keywords:** lungfish, skin, structure, immunolabeling, ultrastructure, evolution

## Abstract

Here we report the immunolocalization of mucin, nestin, elastin and three glycoproteins involved in tissue mineralization in small and large juveniles of *Neoceratodus forsteri*. Both small and larger juvenile epidermis are mucogenic and contain a diffuse immunolabeling for nestin. Sparse PCNA-labeled cells, indicating proliferation, are found in basal and suprabasal epidermal layers. No scales are formed in small juveniles but are present in a 5 cm long juvenile and in larger juveniles. Elastin and a mineralizing matrix are localized underneath the basement membrane of the tail epidermis where lepidotriches are forming. The latter appears as “circular bodies” in cross sections and are made of elongated cells surrounding a central amorphous area containing collagen and elastin-like proteins that undergo calcification as evidenced using the von Kossa staining. However, the first calcification sites are the coniform teeth of the small juveniles of 2–3 cm in length. In the superficial dermis of juveniles (16–26 cm in length) where scales are formed, the spinulated outer bony layer (squamulin) of the elasmoid scales contains osteonectin, alkaline phosphatase, osteopontin, and calcium deposits that are instead absent in the underlying layer of elasmodin. In particular, these glycoproteins are localized along the scale margin in juveniles where scales grow, as indicated by the presence of PCNA-labeled cells (proliferating). These observations suggest a continuous deposition of new bone during the growth of the scales, possibly under the action of these mineralizing glycoproteins, like in the endoskeleton of terrestrial vertebrates.

## 1. Introduction

The skin of fishes is made of a variably thick epidermis while the dermal component differentiates new scales [1,2,3,4,5,6]. After development and larval stages, the epidermis of fish becomes variably stratified and, aside from the prevalent keratinocytes, contains numerous other cell types [1,2,7,8]. During the development of fish skin, the dermis becomes organized into two main layers, a looser external layer and a more compact inner dermal layer, richer in extracellular fibrils. In most extant fish between the two dermal layers, dermal scales differentiate and eventually form a bony outer or limiting layer and an inner fibrous layer called elasmodin [3,9,10]. The mechanically resistant scales, called ELASMOIDS are utilized for specific adaptations and they include osteogenic cells. Elasmodin is a fibrous connective tissue made of crisscrossed and dense collagen fibrils poorly or not calcified. Instead, the more external or limiting layer of elasmoid scales is calcified and is forming a series of bony spinules over most or part of the scale.

In the integument of fishes, the evolution of a dermal skeleton made of scales provides the prevalent protection from the liquid environment since the epidermis remains soft and does not generate a corneous layer. It has been reported that neural crest cells migrate into the dermis of numerous fish and at later stages, these osteogenic cells are at the origin of dermal bones although this is not specifically known for the Australian lungfish *Neoceratodus forsteri* [3,10,11]. However, other more recent studies have challenged previous information, indicating that dermal scales derive from mesodermal and not from neural crest cells [12,13]. The scales of most extant fish have lost the thickness of those present in their progenitors from the Devonian and Carboniferous periods, including those of the dipnoans, presently considered in a key position for explaining the evolution of tetrapods [14].

In particular, in the Australian lungfish, *Neoceratodus forsteri*, the development, morphology and ultrastructure of the epidermis and scales have been studied in comparison to the scales of actinopterygians [7,11,15,16]. Like other elasmoid scales, those of *N. forsteri* are made of two main layers: a calcified outer layer indicated as squamulin and an inner fibrous layer of elasmodin. The morphological study indicates that scale growth occurs mainly along the margins of the scales but it is unknown whether mineralizing glycoproteins like those of bones in terrestrial vertebrates are also involved in the process, such as osteonectin, osteopontin, and alkaline phosphatase. These proteins are involved in the regulation of calcium apatite deposition onto fibrils of collagen of type I [17,18]. A study on the genome of a teleost fish reported the presence of osteopontin in bones and also in dermal scales [19]. However, the latter study did not evaluate the cellular tissue expression of the gene and the localization of its coded protein in bony tissues. Also, a study on another species of teleost showed the localization of alkaline phosphatase and osteopontin in various tissues of the regenerating fin and osteocalcin and chondroitin sulphate in the mineralizing of the regenerating lepidotrichia of the pectoral fin [20]. Although previous morphological studies have shown the formation and microscopic structure of *N. forsteri* scales, no data are currently available on the presence and localization of some of the main mineralizing glycoproteins known in the bones of tetrapods. The present immunohistochemical study mainly aims to determine whether the epidermis of small juveniles of *N. forsteri* produces mucine-like proteins like in later stages. Also, the study aims to detect and localize some mineralizing glycoproteins in the growing scales of the Australian lungfish in order to evaluate whether they may also be involved in scale mineralization. The study also shows that the first mineralization of small lungfish occurs in the teeth.

## 2. Materials and Methods

### 2.1. Animal Samples

The present study was conducted on a total of 8 specimens that were classified according to a previous staging system [15,21]. The biological material was initially derived from four individuals at various stages that included small juveniles of 2.5 cm (whole body, around stage 50) and three juveniles of 16, 19.5, and 26 cm total length (stages 60 to sub-adults). Small skin samples from the initial four specimens were collected in a ventral area post-cloaca (details in [22]). In the other three specimens of small size, 2.0–3.0 cm in length, the skin was instead collected from the entire body, head, trunk, and tail areas. Finally, in one juvenile of 5 cm in length (about 5 months post hatching), only half of the tail was utilized. The skin from all the 8 specimens was utilized for histology and immunolabeling as reported below.

### 2.2. Methods and Preparation for Microscopy

Briefly, some tissues in all 8 specimens were fixed in Carnoy fixative while others from the same specimens were instead fixed in 4% paraformaldehyde in neutral phosphate buffer, dehydrated, and embedded in Lowcryl 4M or in JB4 hydrophilic resins for immunohistochemistry. These tissues were sectioned in longitudinal or cross sections with an ultramicrotome at 1–4 μm thickness and, for histology, some sections were stained with 0.5% toluidine blue. Some other sections were also reacted with Periodic acid and then with the Shiff reactive for detecting glycoproteins (PAS histochemical reaction, counterstained with methylene blue).

For immunohistochemistry, the following mouse antibodies were used on tissues of the initial four specimens: anti-osteonectin (AON-1, Termine JDNIDR from NIH, DSHB, Iowa City, IA, USA), anti-osteopontin (MPIIIB10, Solursh M., Franzer A., University of Iowa, DSHB, USA), and anti-alkaline phosphatase (B4–78-s, Katzmann JA, Majo Clinic, Rochester, NY, USA, DSHB, USA). For the detection of elastin, a rabbit anti-elastin antibody (ab21610, Abcam, Cambridge, UK) was utilized, and for mucin, a goat anti-mucin antibody (C-20, Santa Cruz Biotech, Santa Cruz, CA, USA). A mouse antibody against rat nestin (rat-401, DSHB, USA) was also utilized. Finally, a rabbit PCNA antibody (GTX100539, GeneTex, Irvine, CA, USA) was employed to detect sites of cell proliferation within the skin.

Tissue sections of 2–4 μm thickness were collected using an ultramicrotome and were incubated overnight at 4 °C with a 1:50–200 concentration (*v*/*v*) of the antibodies in 0.1 M phosphate buffered solution at neutral pH containing 2% BSA. In control sections, the primary antibodies were omitted. After rinsing in buffer, the sections were incubated for 1 h at room temperature with secondary antibodies (1:200 dilution in buffer) against mouse, rabbit, or goat IgGs according to the primary antibody utilized, and conjugated with FITC (green) or TRITC (red). Finally, the sections were counterstained with the nuclear fluorophore DAPI (blue). Examination of the sections was conducted using an epifluorescence microscope with proper filters for the three fluorophores here utilized. Images were collected by a digital camera and the digitalized images were used to compose the figures using Adobe Photoshop, version 8.0.

### 2.3. Bioinformatics Control

Since the studied proteins are not annotated for the genome of *N. forsteri* [23], the amino acidic sequences hit by the employed antibodies were compared wherever possible, since the epitope of some of the utilized antibodies was not indicated or the entire protein was the antigen. Comparison was checked using the BLAST server at https://blast.ncbi.nlm.nih.gov/Blast.cgi?PAGE=Proteins (accessed on 1 June 2023) between the antigens tagged by the antibodies with those of orthologous proteins present in a closely related sarcopterygian lungfish (*Protopterus annectens*) to *N. forsteri*, and with other fish and amphibian proteins.

## 3. Results

### 3.1. Bioinformatics Control

The preliminary control was conducted in order to detect the most conserved sequences or epitopes present in orthologous fish proteins. The identified proteins showed a large identity (sequence correspondence) with that of proteins that served to produce the employed antibodies. Nestine and elastine with numerous common epitopes were found in *P. annectens* (support Figures S1 and S2). A check-up of *P. annectens* genome indicated that at least two genes with their coded proteins for osteonectin and alkaline phosphatase are present (Figure S3). Instead, ostepontine was not detected in the genome of any lungfishes (*Protopterus* and *Lepidosiren*). Whether this results represents a true lack of the gene in these species or it derives from incomplete genome sequencing remains to be determined. The bioinformatics data indicate that the employed antibodies should cross-react with similar proteins present in some actinopterigian fish and likely also with the Australian lungfish.

### 3.2. Skin Histology and Fine Cytology in Growing Juveniles

**Figure 1:**
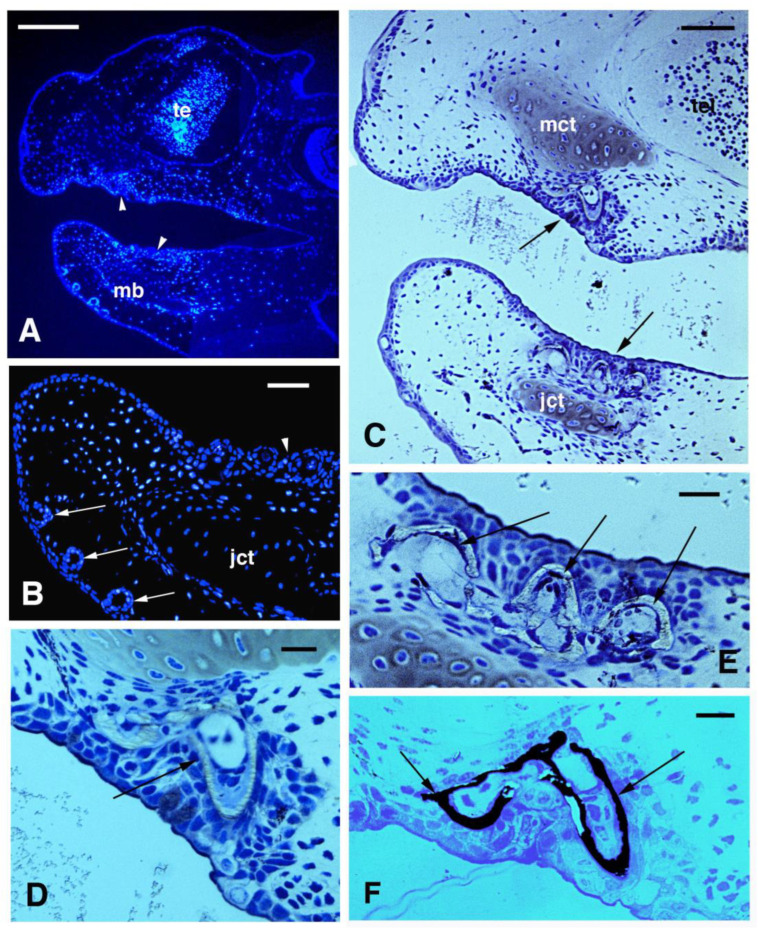
DAPI immunofluorescence (**A**,**B**) and histology (**C**–**F**, toluidine blue stain) of the skin and oral epithelium of the head in a 2.5 cm long larva. (**A**) head featuring a thin epidermis and thicker oral epithelium (arrowheads). Bar, 100 μm. (**B**) Close up to the tip of the jaw showing the invaginating teeth epithelium (arrowhead) and forming epithelial ampullae (sensory organs). Bar, 20 μm. (**C**) particular of the forming teeth (arrows) in the maxilla and jaw. Bar, 50 μm. (**D**) Close up on a pointed tooth (arrow) with mineralized layer (enamel). Bar, 10 μm. (**E**) three forming and mineralized teeth (arrows) in the jaw. Bar, 10 μm. (**F**) Dark precipitate of calcium (arrows) in the mineralized layer of two teeth in the maxilla. Bar, 10 m. **Legends**: jct, cartilage of the jaw (Mekel); mb, mandible (jaw); mct, cartilage of the maxilla (palatoquadrate); te, telencephalon.

**Figure 2:**
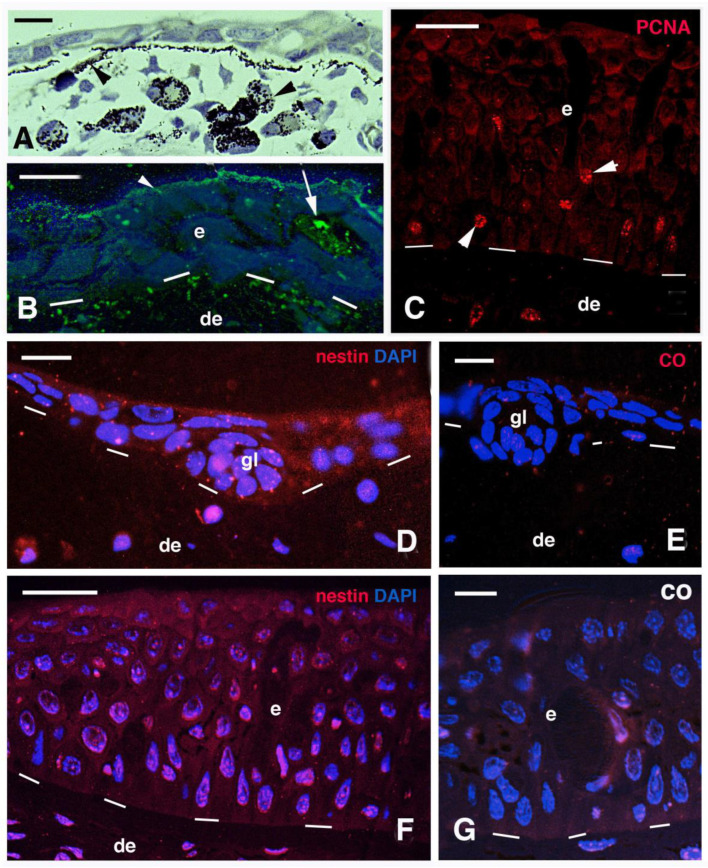
Histology (**A**) and immunofluorescence (**B**–**G**) of small and larger juvenile skin. (**A**) Thin epidermis with accumulating melanophores (arrowheads) in the dermis. Bar, 10 μm. (**B**) Small juvenile skin with external mucin immunoreactivity (arrowhead) and a mucous cell (arrow). Mucin-reactive dots are also seen along the basement membrane, suggesting presence of mucine-like glycoproteins. Bar, 10 μm Bar, 10 μm. (**C**) Thick epidermis of large juvenile containing PCNA-labeled nuclei (arrowheads). Bar, 20 μm. (**D**) diffuse nestine immunofluorescence in keratinocytes of small fish epidermis. Bar, 10 μm. (**E**) Immunonegative section. Bar, 10 μm. (**F**) Weak nestin immunofluorescence in thick epidermis of larger juvenile fish. Bar, 20 μm. (**G**) Negative control section of larger juvenile epidermis. Bar, 10 μm. **Legends**: de, dermis; e, epidermis; gl, likely forming gland. Dashes underline the epidermis.

The general epidermis of the head, trunk, and tail in juveniles of 2.5–3.0 cm in length (stages 50–52) showed 2–3 cell layers with sparse mucous cells among keratinocytes. In the skin of the head, the epidermis on both maxilla and mandible sides formed pear-like invaginations of 10–20 μm in size, probably representing glandular or sensorial organs (Figure 1A,B). In the oral cavity, pointed teeth were forming below the epithelium of both maxilla and jaw, and thin layers of enamel and dentine were already deposited as also indicated by the Von Kossa reaction for calcium (Figure 1C–E). In the trunk skin, mesenchyme melanophores with melanin granules (melanosomes) were accumulating underneath the bi-stratified epidermis (Figure 2A).

The epidermis of these small juveniles showed a granular immunolabeling for mucin, and often a superficial immunolabeled layer of mucus secretion was observed (Figure 2B). Sparse PCNA-labeled cells were seen in the epidermis of small and larger juveniles, indicating a continuous cell proliferation, prevalently in the basal layer (Figure 2C). Diffuse immunolabeling for nestin was seen in a small and larger juvenile epidermis while the labeling was absent in controls (Figure 2D–G). Beneath the cubic- or polygonal-shaped cells forming the basal layer of the epidermis, few mesenchymal cells were present. Underneath the epidermis, mesenchymal cells were associated with a denser stratum of extracellular fibrils of the basement membrane and with pigment cells. In other regions of the posterior trunk and tail, a dense connective layer of 5–7 μm contains flat fibrocytes covering a loose connective tissue.

**Figure 3:**
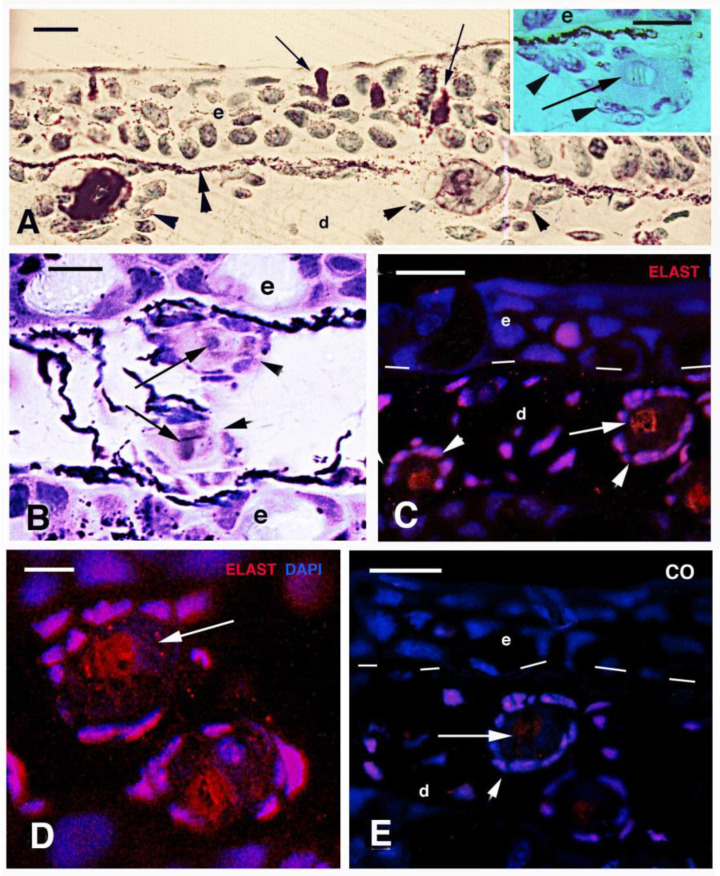
Histology (**A**,**B**) and immunolabeling (**C**–**E**) of the skin in *N. forsteri* specimen 5 cm long. (**A**) Cross-section of multistratified tail epidermis showing sparse mucous- secreting cells (arrows) using toluidine blue stain. Arrowheads indicate cells surrounding “circular bodies” located beneath the basement membrane, bordered by the cytoplasmic elongation of pigment cells (double arrowhead). Bar, 20 μm. The inset (bar, 10 μm) shows the numerous fibroblasts (osteoblasts, arrowheads) surrounding the amorphous material and the central, pale region (arrow). (**B**) Close up on two “circular bodies” after PAS-methylene blue staining. The central region of the bodies is blue (arrow) while fibroblasts (arrowheads) surround a weakly pink (PAS-positive) fibrous material. Bar, 10 μm. (**C**) Dermal fibroblasts (arrowheads) surrounding circular bodies containing a central region immunoreactive for elastin (arrow). Bar, 20 μm. (**D**) Higher magnification of two circular bodies with central elastin immunostaining (arrow). Bar, 10 μm. (**E**) immunonegative control (arrow). Bar, 20 μm. **Legends**: d, dermis; e, epidermis. Dashes underline the epidermis.

In cross sections of the tail region of a 5 cm long juvenile (5–6 months old, stages 55–56) at intervals of 70–100 μm beneath the epidermis, groups of connective cells were concentrically arranged forming “circular bodies” of 15–20 μm in diameter. Their cells, possibly osteogenic fibroblasts (osteoblasts), surrounded a central amorphous extracellular material of 10–20 μm in diameter (arrowheads in Figure 3A and inset). The circular bodies contained a paler core (arrows in Figure 3A inset, B) surrounded by an unstained amorphous material using toluidine blue (acidophilic, see arrowheads in Figure 3A inset). This amorphous material showed a weak or variable staining using the PAS reaction while the central core showed affinity for toluidine blue (basophil). After immunolabeling for elastin, thin elastin-containing fibrils appeared localized underneath the epidermis but larger elastin immunolabeled micro-areas of 5–10 μm surrounded by dermal cells of the “circular bodies” (Figure 3C,D). The latter were mainly localized along the basement membrane of the epidermis and the immunostaining appears specific in comparison to controls (Figure 3E).

**Figure 4:**
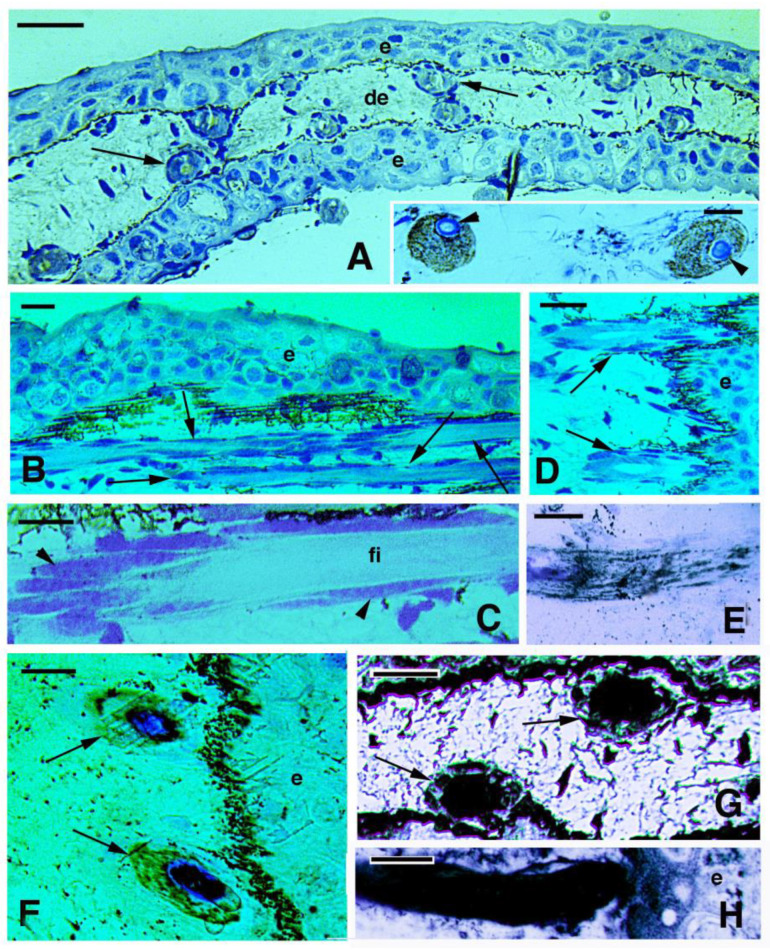
Histology (toluidine blue) and von Kossa histochemistry (dark) of the tail skin in a 5 cm long larva. (**A**) Cross section showing the two sides of the epidermis with the “circular bodies” (lepidotrichia) located underneath. Bar, 20 μm. The inset (bar, 10 μm) shows a dark granulation derived from the von Kossa reaction for calcium. The central cores (arrowheads) are uncalcified. (**B**) Longitudinal section showing two lepidotrichia associated with numerous osteoblasts (arrows) underneath the epidermis. Bar, 10 μm. (**C**) Detail of a lepidotrichium with osteoblasts (arrowheads) surrounding the pale fibrous region (fi). Bar, 10 μm. (**D**) two terminal lepidotriches tips (arrows) contacting the epidermis. Bar, 10 μm. (**E**) longitudinal section of weakly mineralized (dark areas) lepidotrichium. Bar, 10 μm. (**F**) Two tips of weakly mineralized lepidotriches (arrows) terminating on the epidermis. Bar, 10 μm. (**G**) two heavily calcified lepidotriches (arrows) in cross section (roundish bodied). Bar, 10 μm. (**H**) A longitudinal section of a heavily calcified lepidotrichium. Bar, 10 μm. **Legends**: de, dermis; e, epidermis; fi, fibrous component of the lepidotrichia.

In the cross-sectioned tail fin, the “circular bodies” appeared opposed or alternated along the dermis, and they were variably calcified using the von Kossa method (Figure 4A and inset). When the same tail of the 5 cm long specimen was sectioned longitudinally, the “circular bodies” appeared as long fibrous rods surrounded by fibroblasts, revealing that they are actually mineralizing lepidotriches of the tail fin (Figure 4B–D). Various degrees of calcification, as revealed by the von Kossa reaction, were present in the fibrous amorphous matrix forming the lepidotriches, from a diffuse but low calcium impregnation to an intense calcification (Figure 4E–H). However, Their central core remained uncalcified and still basophil (toluidine positive; Figure 4F).

### 3.3. Scale Histology and Immunofluorescence

**Figure 5:**
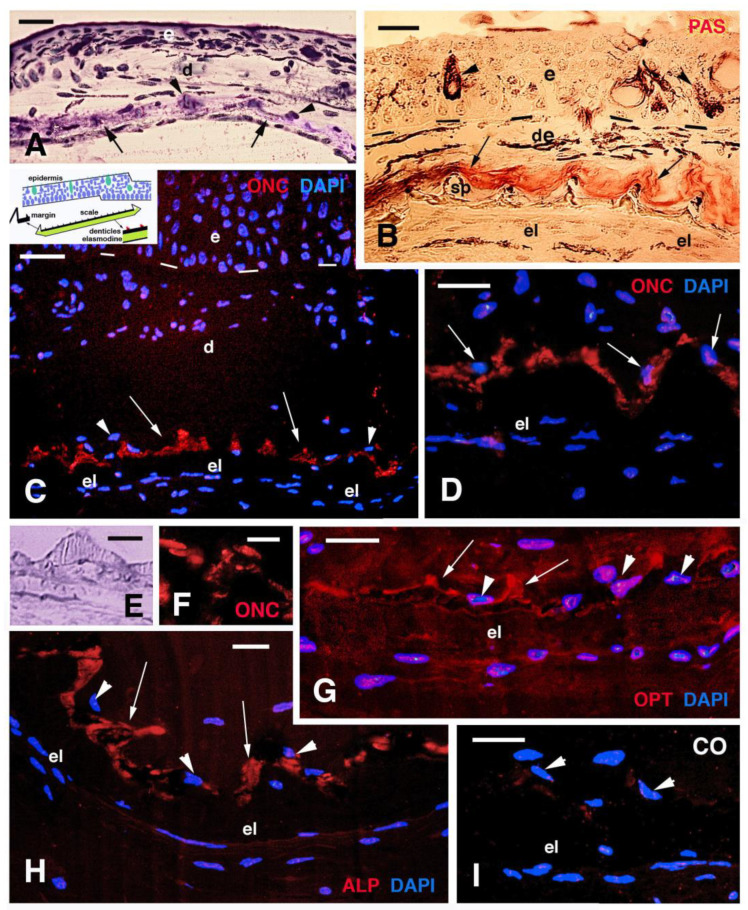
Histological sections of the skin of 5 cm long specimen (**A**) and of juveniles’ (**B**–**I**) skin. (**A**) Histological section showing a thin epidermis, loose dermis, spinulae (arrowheads), and the underlying thin fibrous layer (elasmodin, arrows) of the scale. Toluidine blue stain. Bar, 20 μm. (**B**) PAS-stained section showing intensely reactive mucus cells (arrowheads) and numerous melanophores in the dermis. The spinulae of the outer layer (squamulina) are surrounded by PAS-positive material (arrows). Bar, 20 μm. (**C**) Spinulated layer (denticles, arrows) immunolabeled for osteonectin (ONC). Arrowheads indicate cells associated to the denticles. The indicative position of dermal scales is shown in the associated drawing. Bar, 20 μm. (**D**) Detail of bony denticles immunolabeled for osteonectin and the associated cells (arrows indicating the nuclei). Bar, 10 μm. (**E**) detail on one bony denticle. Toluidine blue stain. Bar, 5 μm. (**F**) immunolabeled denticle for osteonectin (only TRITC immunofluorescence). Bar, 5 μm. (**G**) Osteopontin (OPT) labeling of denticles (arrows). A weaker immunofluorescence is also seen in the surrounding connective tissue and in the elasmodin layer of the scale. Arrowheads indicate cells associated to the denticles. Bar, 10 mm. (**H**) Immunolabeling of the spinulated layer (arrows) for alkaline phosphatase (ALP). Arrowheads indicate the nuclei of associated cells. Bar, 10 μm. (**I**) immunonegative control section (CO) with nuclei of the cells (arrowheads) associated to the spinulae. Bar, 10 μm. **Legends**: de, dermis; e, epidermis; el, elasmodin (layer); sp, spinulae or denticles (squamulin).

The progressive stages of scale morphogenesis that were previously described (Kemp, 1987; Kemp et al., 2015 [16,21]) were not available in our limited sampling and therefore a successive series of developing scales could not be observed. Scales were absent in the four small juveniles (stages around 50) while they were forming in the 5–6-month-old specimen of about 5 cm in length, and were well formed in the superficial dermis of the three larger juveniles indicated above. Scales were present as discrete units underneath the epidermis of juveniles 16 cm long (stages 58–60 and sub-adults according to Kemp, 1987, 2014). At these stages, fishes have already formed a spinulated or denticulated outer layer (limiting layer, sensu Sire et al. [3]) that comprises squamulin in calcification (sensu Kemp et al. [16]) and is located beneath the loose dermis (Figure 5A). The inner layer of the scale, made of fibrous elasmodin, is made of flat fibroblasts mixed to large extracellular collagen fibrils, and is still relatively thin (Figure 5A). In juveniles of 19.5 and 26 cm (sub-adults stage), under the epidermis, the scale appeared thicker in both the external bony layer (squamulin) and the internal fibrous layer (elasmodin), indicating that scales are growing in thickness and in diameter. After staining with PAS for carbohydrates and glycoproteins, aside from the numerous intensely stained mucous cells localized in the epidermis, the loose connective tissue surrounding the spinulae of the mineralized (limiting) layer also appears stained (Figure 5B). This region of loose connective tissue present on the top of the scale contains a few flat cells and their nuclei that also contact the bony spinulae (arrows in Figure 5C,D).

The immunodetection of osteonectin showed that the spinulae, or part of them, contain this protein (Figure 5C,D). The immunolabeling was mainly localized on the spinulated surface of the bony layer, likely associated with the flat cells covering or in contact with the spinulae (Figure 5D–F). Osteonectin immunolabeling was particularly intense in the external border (margins) of scales (indicated with an arrow in the drawing of Figure 5C). The immunoreaction for osteopontin revealed an intense reaction on the spinulae but was also diffuse in the PAS-positive connective tissue located above the spinulae and in the elasmodin layer located underneath the spinulae (Figure 5G). Finally, the immunoreaction for alkaline phosphatase was similar to that for osteonectin, and was detected only along the spinulated mineral layer (Figure 5H). In control sections, a weak to absent fluorescence is detected (Figure 5I). In summary, the immunolabeling for osteonectin (and alkaline phosphatase and osteopontin) was mainly localized on the spinulated surface of the bony layer, likely associated with the flat cells covering or in contact with the spinulae (arrowheads in Figure 5C–H). The available scanty biological material did not allow to follow further the calcification process and bony formation in scales of *N. forsteri*.

**Figure 6:**
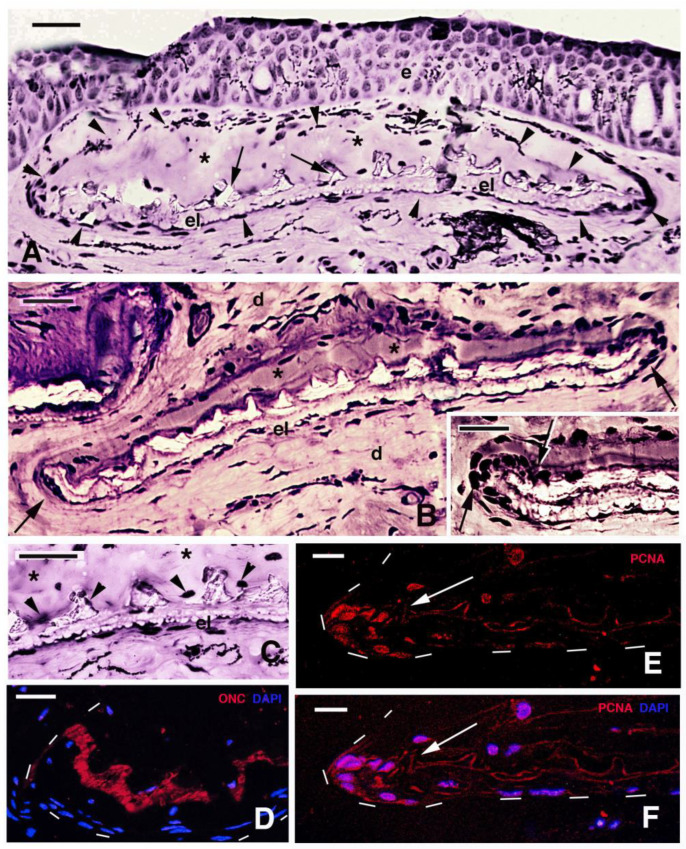
Histology (**A**–**C**) and immunofluorescence (**D**,**F**) of juvenile skin. (**A**) juvenile of 16 cm featuring an entire scale (arrows) located underneath the epidermis. Arrows indicate the outer or limiting layer formed by bony spinulae. Asterisks indicate a pocket-like area (arrowheads) occupied by loose dermal connective tissue. Bar, 20 μm. (**B**) Elasmoid scale with its definitive tilted disposition in the dermis. Asterisks indicate the loose dermal pocket associated with the spinulated bony surface. The arrows indicate the marginal regions (anterior, right, and posterior on the left) of the scale. Bar, 30 μm. The inset (bar, 20 μm) shows a detail of the cells (arrow) accumulated at the margin of the scale. (**C**) Detail of central region of a scale showing nuclei of cells (arrowheads) associated with the bony denticles of the scale. Asterisks label the soft dermal layer of the scale pocket. Bar, 20 μm. (**D**) Intense immunolabeling for osteonectin (arrows) in the spinulated layer located at the margin of the scale (dashes). Bar, 20 μm. (**E**) PCNA- immunolabeled cells (nuclei, arrow) located at the margin of a scale (dashed). Bar, 10 μm). (**F**) same section with merged fluorescence for DAPI (double- labeling pink nuclei, arrow). Bar, 10 μm. **Legends**: d, dermis; e, epidermis; el, elasmodin (fibrous basal layer of the scale); sp, bony spinulae (denticles) of the outer layer of the scale.

In juvenile skin, each scale appeared located within a connective tissue pocket or capsula (arrowheads in Figure 6A), made of an upper loose or lacunar dermis contacting the pigmented layer present at the base of the epidermis, while the scale was present underneath (asterisks in Figure 6A). The extremities or margins of these scales were upfolded (arrows in Figure 6B), and their fibrous layer terminated, undistinguished, in the loose connective tissues separating the scales one from another and in continuity with the dermis. The extremities of these scales were upfolded and their fibrous layer terminated, un-distinguished, in the loose connective tissues separating the scales one from another and reaching the base of the epidermis where numerous melanophores were localized (Figure 6A,B). Above the bony spinulae of squamulin, a loose connective lacunar tissue containing a few cells was present (asterisks in Figure 6A–C). The scale margins, where osteonectin is present (Figure 6D), contained more numerous and likely osteogenic cells (scleroblasts) than in more central regions of the scale, as also shown by their PCNA labeling (Figure 6E,F). As a whole, it appeared that the elasmodin layer of scales was in continuity with the surrounding dermis, at least at the stages here observed.

**Figure 7:**
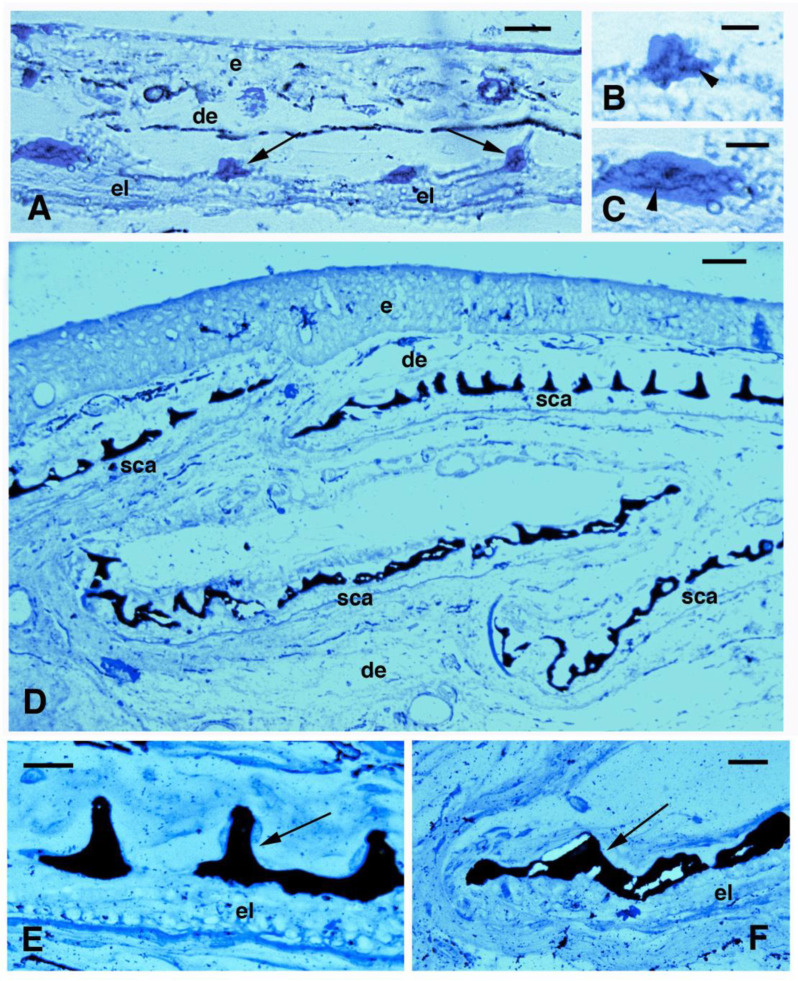
Von Kossa staining for calcium deposition (black) in 5 cm long specimen (**A**–**C**) and juveniles (**D**–**F**). Von Kossa stain (dark) and wekly methylene blue stain. (**A**) Formation of denticles of squamulin (arrows) on a thin scale. Bar, 10 μm. (**B**) Close up of a pointed denticle with initial calcification (dark, arrowhead). Bar, 5 μm; (**C**) Larger denticle with initial calcification (arrowhead). Bar, 5 μm. (**D**) low magnification and panoramic view of a longitudinal section showing four calcified scales in their squamulin (denticles) layer. Bar, 20 μm. (**E**) Close up of a calcified scale (arrow on denticles). Bar, 10 μm. (**F**) detail on the marginal area of a scale with calcified denticles (arrow). Bar, 10 μm. **Legends**: de, dermis; e, epidermis; el, elasmodin; sca, scales.

Using the von Kossa histochemical stain for revealing calcium mineralization, an initial calcification appearing as small dense granules–filaments, was seen inside the denticles (Figure 7A–C). In juveniles, the denticulate bony outer layer (squamulin) instead appeared heavily calcified, from the central region to the peripheral regions of the scale (Figure 7D). However, some small non-mineralized segments were also seen between the mineralized denticles (squamula breaks) resting upon unlabeled elasmodin (Figure 7E). Calcification also extended heavily at the anterior and posterior margin of the scales (Figure 7F).

## 4. Discussion

### 4.1. Epidermal Proteins

The mucogenic nature of the epidermis in the lungfish, secerning mucins that were noted in previous studies on large juveniles and sub-adult lungfishes, is here confirmed also for the epidermis of small juveniles [6,7,24,25,26,27]. For the Australian lungfish, previous studies indicated that after hatching, tiny juvenile fishes of 2–3 cm resemble externally later stages, and that they simply grow without large external morphological changes [21,28]. However, many changes occur in the inner organs of the small fish as it grows, from stages 50 to 60 and beyond, also associated with diet and environmental changes. Like for other fish, processes of metamorphic restructuring occur in the inner organs of this sarcopterigian species as the fish grows [21], and adults are considered as neotenic [29].

As regards the skin, pigmentation changes, the epidermis becomes much thicker with different cell types, and cell proliferation remains active as indicated by PCNA labeling and the presence of the stem cell marker nestin in the epidermis. Nestin appears present, especially in the keratinocytes of the larval epidermis, and this was also observed in other species of bony fishes and amphibians as a cytoskeletal protein of the normal epidermis [6]. Compared to adult keratinocytes, those of the small fish of 2–3 cm in length (stage 50) show that most keratin filaments are free in the cytoplasm, while they rarely form dense bundles like in the adult epidermis, and no corneous layer is formed in the epidermis [7,22,26]. Various types of IFKs (Intermediate Filament Keratins) with a molecular weight (MW) 40–63 kDa, mainly acidic, with a few also neutral and possibly also with a slightly basic keratin are found in the epidermis of the Australian lungfish [30,31].

### 4.2. Teeth and Lepidotriches Calcification

The present study shows that teeth are the first bony organs of the lungfish to undergo mineralization, already seen in small juveniles around stages 49–50 [32]. During the following development, the initially isolated teeth cusps merge into the adult plates. Lepidotriches of the tail appear to originate in association with the epidermis of the tail, but further studies are needed on this point. The central region, which is toluidine positive (Figure 4A inset and F), likely contains an elastin-like protein but this is surrounded with a fibrous material deposited by the surrounding fibro- or osteoblasts. Ultrastructurally, this region appears like the central part of the actinotrichia of teleosts, indicated as elastoidine [33], however, a specific study on this point should be conducted.

Lepidotriches in the tail fin undergo progressive calcification and are largely mineralized by stages 56–58. In actinopterygians, lepidotriches’ mineralization derives from mesenchymal cells of the paraxial mesoderm of the embryo that acquire an osteoblast role [12]. The present study suggests that the central core of lepidotriches contains mainly elastin-like proteins, known to be basophil with pI over 9.5, that might control the rate of mineralization [34] of surrounding tissue of the lepidotrichia. Around this core, mainly acidophilic collagen fibrils (pI 4–5) are present that undergo mineralization to form the lepidotrichia of the tail.

### 4.3. Scale Mineralization

Scales are continuously growing, especially in juveniles and sub-adults. In previous studies, it was indicated that the beginning of scale formation in *N. forsteri* was around stage 53 [11], a stage that was not available in our sampling. In our limited number of samples, while scales were absent in the four small juveniles available in the present study (2–3 cm in length), thin elasmoid scales were well formed in the lungfish of 5 cm around stage 56–58, and thicker scales were present in juveniles of 16–26 cm (late juveniles to sub-adults, [15]). The thickness of scales in adult lungfishes increases, especially on the squamulin or outer bony layer [16]. The basement membrane of the epidermis in small juveniles’ skin contains elastic fibers that likely allow some stretching of the integument. The study shows that this region in the tail mainly contains collagen fibrils and appears the first to be calcified around elastic fibers of the lepidotriches, previously indicated as osteoid material [30] While elastic fibers mineralize with some difficulty later, the process occurs where collagen fibrils are present around the elastin fibers in the small and larger juveniles (see Figure 5 and Figure 6 in [30]).

Like in teleost scales [4,10], the light elasmoid scales of *N. forsteri* also show a denticulate bony layer (limiting or outer layer, sensu Sire et al. [3]) and an inner fibrous elasmoid layer containing elasmodin. In contrast, the lower fibrous layer of elasmoid scales contains sparse fibrocytes but remains not calcified and numerous collagen fibrils are deposited in organized bundles that form a plywood pattern that increases in density and thickness with the growth of the fish (elasmodin [11,15,16]. As elasmoid scales, the mineralizing scales of the lungfish likely resembles those of teleosts, although they derive from different evolutionary lineages [2,3]. The “spines or denticles” of *N. forsteri* derive from the mineralization of a pre-osseous collagen and glycoprotein-rich matrix, and the present study indicates that osteonectin, osteopontin, and alkaline phosphatase are involved in the process. It is known that these molecules determine the regulated precipitation of calcium apatite onto collagen I [17,18], known to be present in squamulin. The localization of osteonectin is likely also associated with the marginal growing region of the scale where osteogenic cells are more numerous and also proliferating (inset of Figure 5B,E,F). These cells, also described inside tubules or cavities of squamulin, are likely involved in bony deposition for the circular growth of scales [11,16]. This finding suggests that osteonectin was already present in the scales of ancient sarcopterygians, the likely ancestors of the tetrapods that later evolved a highly mineralized endoskeleton which also utilizes these glycoproteins for mineralization [28,35]. The lack of osteonectin, alkaline phosphatase, and calcium deposits in the elasmodin layer, noted in this study, confirms that this layer is not mineralized in the Australian lungfish [16].

The presence of osteopontin, here observed by immunohistochemistry in lungfish scales, although apparently absent in the genome of *N. forsteri* [23], is supported by some studies on teleost fish where osteopontin is associated with the mineralization of the skeleton and also in the scales and lepidotriches [19,20]. Detailed information on other proteins involved in the mineralization of *N. forsteri* will become available after a complete protein annotation of the lungfish genome [23].

## 5. Conclusions

The present study shows that cell proliferation remains active mainly in basal but also in suprabasal layers of the mucogenic epidermis of juvenile lungfishes, that the initial sites of calcification are in the teeth, and that calcium phosphate is deposited around an inner core of protein material containing an elastine-like protein in forming tail lepidotriches. Growing scales contain proliferating osteoblasts located along the scale margins and mineralizing glycoproteins, typical of tetrapod-calcifying bones, are implicated in the deposition of calcium phosphate in the expanding scales.

## Data Availability

Not applicable.

## Supplementary Materials

The following supporting information can be downloaded at: https://www.mdpi.com/article/10.3390/jdb11030035/s1. Figure S1: Clustal W comparison between the initial amino acid sequences (aa) of rat versus *P. annectens* nestin; Figure S2: CLUSTAL-W comparison between human (h, AN AAC98395.1) and *Protopterus annectens* (p, AN XP_043910108.1) elastin, showing numerous common epitopes; Figure S3: two examples of Clustal-w selected proteins form the bull (Bos taurus, b, the original antigen of the antibodies here utilized), and the amino acid sequences (n) identified in the genome of *Protopterus annectens* (p).
