# Supplementary material for: Immunolocalization of Some Epidermal Proteins and Glycoproteins in the Growing Skin of the Australian Lungfish (Neoceratodus forsteri)
Source: J Dev Biol. 2023 Aug 14;11(3):35. doi: 10.3390/jdb11030035 (PMC10443291; doi:10.3390/jdb11030035)
Supplement: Supplementary file 1 [file jdb-11-00035-s001.zip › figure S1.pdf]

Comparison between rat nestin (r, P21263, aa 1-326) and nestin from *Protopterus annectens* (p, XP_043935726.1, aa 1-360)

```
r               ------MEGCVGEESFQMWELNRRLEAYLTRVKTLEEQNQLLSAELGGLRAQSGDTSWRA
p               MERSMRRNQTMGTESQQMYELNKRLEGYLSRVKFLEQENELLKEEIQELQLEQSKTSWKN
                       :  :* ** **:***:***.**:*** **::*:**. *:  *: :...***: 

r               RADDELASLRILVDQRWREKLEAEVQRDNLAEELESVAGRCQQVRLARERTVQEAACSRR
p               EYQKELRALRESLDELYRDKSQTELERDRMYDELLLMKDRWQKEKQEQELVKQKVKESKQ
                . :.** :**  :*: :*:* ::*::**.: :**  : .* *: :  :* . *:.  *::

r               ALEAEKNARGWLSTQAAELERELEALRAAHEEERAHLNAQAACAPR--RPPAPPHGSPVR
p               ELEEETRAHNWLKQKAFQLEDELQLLQDAHEEEKLSLQQEVSERSYSFTELRPAHTPAYP
                 ** *..*:.**. :* :** **: *: *****:  *: :.:  .       *.* ..  

r               APEVEDLARRLGEVWRGAVRDYQERVAHMESSLGQARERLSQAVRGARECRLEVQQLQAD
p               AIEVQEYTSQLSEIWKGAVRSYQEEIDQMEASLQDAKDQLQSIAREKKEAYQLLQNLQRE
                * **:: : :*.*:*:****.***.: :**:** :*:::*.. .*  :*.   :*:** :

r               RDSLQERREALEQRLEGRWQDRLQATDKFQLAVEALEQEKQGLQSQIAQILEGGQQLAHL
p               LDSLQVRKEMLEKSVMKHQQEQQDCRHEFQSEIDSMEDEKEELRQQIVQILKDRQKLMEL
                 **** *:* **: :  : *:: :. .:**  ::::*:**: *:.**.***:. *:* .*

r               KMSLSLEVATYRTLLEAENSRLQTPGRGSQASLG-------------------------F
p               KMSLSLEVATYRSLLEAESIRLQPFMEYSPASVIRDTKLDIKSNKPPSLPNNITDVRRKF
                ************:*****. ***.  . * **:                          *
```
