# Supplementary material for: Immunolocalization of Some Epidermal Proteins and Glycoproteins in the Growing Skin of the Australian Lungfish (Neoceratodus forsteri)
Source: J Dev Biol. 2023 Aug 14;11(3):35. doi: 10.3390/jdb11030035 (PMC10443291; doi:10.3390/jdb11030035)
Supplement: Supplementary file 1 [file jdb-11-00035-s001.zip › figure S2.pdf]

Comparison between elastins from huma (h, AAC98395.1) and elastin from *Protopterus annectens* (p, XP_043910108.1)

```
h                MAGLTAAAPRPGVLLLLLSILHPSRPGGVPGAIPGGVPGGVFYPGAGLG------ALGGG
p                --MASCKLLWTLHALLTLCLLRTSLQGGVPGAGAGGLYG-ALYPGGVHGGYKPGKAAGIG
                     :.    .   ** *.:*:.*  ****** .**: * .:***.  *      * * *

h                ALGPGGKPLKPVPGGLAGAGLGAG-LGAFP---------AVTFPGALVPGGVADAAAAYK
p                AGGLGAGGLGAIPGAGVGTGIGAGGLGVIPGAGLGTGLGAGVGAGGLIPGLAAGTGIGVK
                 * * *.  * .:**. .*:*:*** **.:*         * . .*.*:** .*.:. . *

h                AAKAGAGLGGVPGVGGLG------------------------------------------
p                PPKLGAGIGGLGGLGGVPGGLGGLGGVPGGLGGFGGVPGGLGGFGGIPGGLGGLGGIPGG
                 ..* ***:**: *:**:

h                ------------------------------------------------------------
p                LGGLGGIPGGLGGLGGIPGGLGGLGGLGVGQYPGGVKPPKVPGTGVGVPGTGVGGTGIGG
h                --------VSAGAVVPQP------GAGVKPGK-VPGVGLPGVYPGGVLPG----ARFPGV
p                VGTGVQPGVSSGAVIPQTGAQPGVGAAGKPGKQLPGVGIPGVYPGGFIPGPGGLLPGTGV
                         **:***:**.      **. **** :****:*******.:**       .**

h                GVLPGVPTGAGVKPKAPGVGGAFAGIPGVGPFGGPQPGVPLGYPIKAPKLPGGYGLPYTT
p                GVLPGVPTGTGIKQKVPGAGGTVAGVPGVGGFGGQQPGVPLGYPIKTPKLPGGYGLPYSS
                 *********:*:* *.**.**:.**:**** *** ***********:***********::

h                GKLPYGYGPGGVAG---AAGKAGYPTGTGVGPQAAAAAAAKAAAKFGAGAAGVLPGVGGA
p                GKLPYGFGTGAGIGGVPGAGLPGFPTGTGAGAQAAAAKAAAKAAKYGAGGR--YPGVG--
                 ******:*.*.  *   .** .*:*****.*.***** **  ***:***.    ****

h                GVPGVPGAIPGIGGIAGVGTPAAAAAAAAAAKAAKYGAAAGLVPGGPGFGPGVVGVPG--
p                SIGGVPGLVPGVG--LGGGLVPGAGAGGRYPGVGSIGGVPGLVPGVGLGGGLVPGAVPGA
                 .: **** :**:*   * *  ..*.*..  . ... *...*****    *  * *.

h                ----AGVPGVGVPGAGIPVVPGAGIPG--AAVPGVVSPEAAAKAAAKAAKYGARPGVGVG
p                VSAKAAKYGYGIPGAGVAGLPGVVPGAGVGRLPGFVPGVGVVPGAGVVPGTGIVPGAGIG
                     *.  * *:****:. :**.   .  . :**.*.  ... .*. ..  *  **.*:*

h                GIPTYGVGAGGFPGFGVGVGGIPGVAGVPSVGGVPGVGGVPGVGISPEAQAAAAAKAAKY
p                ISPKAGVVPGAGIGVSPGAGVVPGTGVGVVPGTGVGVVPGTGVGVVPGTGVGVVPGTGIG
                   *. ** .*.  *.. *.* :**..     *   **   .***: * : ..... :.

h                GVG--TPAAAAAKAAAKAAQFGLVPGVGVAPGVGVAPGVGVAPGVGLAPGVGVAPGVGVA
p                VVPGTGVGAVPGAGLGVSPKAGVVPGVGVVPGAGVGIGS-VIPGVGVVPGVGVVPGAGVV
                  *     .*... . . :.: *:******.**.**. *  * ****:.*****.**.**.

h                PGVGVAPGIGPGGVAAAAKSAAKVAAKAQLRAAAGLGAGIPGLGVGVGVPGLGVGAGVPG
p                PGVGVVPGTGIGVVPGTGVGAVPGAGVGVSPKAVLP--VQPGVLVQPSGTVTSVSGATPA
                 *****.** * * *..:. .*.  *. .    *.      **: *  . .  .*....*.
h                LGVGAGVPGFGAGADEGVRRSLSPELREGDPSSSQHLPSTPSSPRVPGALAAAKAAKYGA
p                PGAANGTVIHGGVAGTGVVQPGVGVVQPG-VGGVAPGAVVPGAGVGVGGVGVGGVGAGKP
                  *.. *.  .*. *. ** :.    :: *  ..    . .*.:    *.:... ..   .

h                AVPGVLGGLGALGGVGIPGGVVGAGPAAAAAAAKAAAKAAQFGLVGAAGLGGLGVGGLG-
p                LKPGAAG-LPFVGGAGLQPGAGLQLPTGIGAGLKPGKIGAGVGLGGVPGVPGAGLGGLGG
                   **. * *  :**.*:  *.    *:. .*. *..  .* .** *..*: * *:****

h                VPGVGGLGGIPPAAAAKAAKYGAAGLGGVLGGAGQFPLG---GVAARPGFGLSPIFPGGA
p                VPGGAGVGGVGVPGVPGAGIGGLGYPAGARYPGGIKPPKPGYGAGVLPGFGYSPIAPGLA
                 *** .*:**:  .... *.  * .  .*.   .*  *     *... **** *** ** *

h                CLG------------------------------KACGRKRK
p                SQLGYPAGKPPKSYGGALNLLAAYRGGYPCGAGKLCGKRK-
                 .                                * **:::
```
