# Supplementary material for: Immunolocalization of Some Epidermal Proteins and Glycoproteins in the Growing Skin of the Australian Lungfish (Neoceratodus forsteri)
Source: J Dev Biol. 2023 Aug 14;11(3):35. doi: 10.3390/jdb11030035 (PMC10443291; doi:10.3390/jdb11030035)
Supplement: Supplementary file 1 [file jdb-11-00035-s001.zip › figure S3.pdf]

A) Comparison Osteonectin in *Bos taurus* (b, NP_776889.1) and oesteonectin in *Protopterus annectens* (p, XP_43925350)

```
b        MRAWIFFLLCLAGRALAAP----------QQEALPDETEVVEETVAEVAEVPVGANPVQV
p        MRVFLLLFFVLVSKTLAVPPIDVQEEAVFDTDQVVLDEPAAEEEVVEIEEPTVGANPVQV
         **.:::::: *..::**.*          : : :  :  ..** *.*: * .********

b        EVGEFDDGAEETEEEVVAENPCQNHHCKHGKVCELDENNTPMCVCQDPTSCPAPIGEFEK
p        ETDDFEE-PEETVEEVVAENPCLNHYCKHGKVCEVDENNMPMCVCQDPSTCPTATGEFEH
         *..:*:: .*** ********* **:********:**** ********::**:. ****:

b        VCSNDNKTFDSSCHFFATKCTLEGTKKGHKLHLDYIGPCKYIPPCLDSELTEFPLRMRDW
p        VCGNDNKTYDSSCHFFATKCTLEGTKKGHKLHLDYIGPCKYIPPCMDNELSEFPFRMRDW
         **.*****:************************************:*.**:***:*****

b        LKNVLVTLYERDEDNNLLTEKQKLRVKKIHENEKRLEAGCDHPVELLARDFEKNYNMYIF
p        LKNVLVTMFERDEENNLLTEKQKLRVKKIFENEKRLSAG-DHSIDLLTLDFVKNYNMYIF
         *******::****:***************.******.** **.::**: ** ********

b        PVHWQFGQLDQHPIDGYLSHTELAPLRAPLIPMEHCTTRFFETCDLDNDKYIALDEWAGC
p        PVHWQFGQLDQHPVDGYLSHTELAPLRAPLIPMEHCTTRFFTECDTDSDRYIALEEWAAC
         *************:***************************  ** *.*:****:***.*

b        FGIKEKDIDKDLVI
p        FGIKEQDIDKNLVI
         *****:****:***
```

B) Comparison between Human Alkaline phoshatase (AAB59378.1) and Alkaline phosphatae in *Protopterus annectens* (p, XP_043926613.1)

```
h        MISPFLVLAIGTCLTNSLVPEKEKDPKYWRDQAQETLKYALELQKLNTNVAKNVIMFLGD
p        ------------------------------------------------------------


h        GMGVSTVTAARILKGQLHHNPGEETRLEMDKFPFVALSKTYNTNAQVPDSAGTATAYLCG
p        -MGVTTVTAARILKGQLEGQSGEEGSLTMDTFPNIALSKTYNVDYQVPDSAGTATAYLCG
          ***:************. :.***  * **.** :*******.: ***************

h        VKANEGTVGVSAATERSRCNTTQGNEVTSILRWAKDAGKSVGIVTTTRVNHATPSAAYAH
p        VKGNYGTIGLNAKAKRYQCSTSKGNEVTSILKRAKDAGKSVGIVTTTRVQHASPAGAYAH
         **.* **:*:.* ::* :*.*::********: ****************:**:*:.****

h        SADRDWYSDNEMPPEALSQGCKDIAYQLMHNIRDIDVIMGGGRKYMYPKNKTDVEYESDE
p        ISDRDWYGDADMPADAISSGCKDIAYQLVHNG-DINVILGGGRKYMFPAGTPDPEYPSES
          :*****.* :**.:*:*.*********:**  **:**:*******:* ...* ** *:.

h        KARGTRLDGLDLVDTWKSFKPRYKHSHFIWNRTELLTLDPHNVDYLLGLFEPGDMQYELN
p        SKAATRKDNRTLVNDWLSSR---KKAVYVWKKADFDAVDESTTDYLMGLFEPGDMKYELN
         .  .** *.  **: * * :   *:: ::*::::: ::*  ..***:********:****

h        RNNVTDPSLSEMVVVAIQILRKNPKGFFLLVEGGRIDHGHHEGKAKQALHEAVEMDRAIG
p        RDKTMDPSIVEMTEKAIKILHKNPKGFFLFVEGGRIDHGHHDTKAKMALTEAVLFDQAIK
         *::. ***: **.  **:**:********:***********: *** ** *** :*:**

h        QAGSLTSSEDTLTVVTADHSHVFTFGGYTPRGNSIFGLAPMLSDTDKKPFTAILYGNGPG
p        RASELTSESDTLTVVTADHSHVFTFGGYTVRGNPIFGLASSVAS-DKKNFTSILYGNGPG
         :*..***..******************** ***.*****. ::. *** **:********

h        YKVVGGERENVSMVDYAHNNYQAQSAVPLRHETHGGEDVAVFSKGPMAHLLHGVHEQNYV
p        YANTSEGRPNVNANTGENKDYMQQTAVPLDSETHGGEDVAIFSKGPMAHLLHGVQEQSYI
         *  ..  * **.     :::*  *:****  *********:*************:**.*:

h        PHVMAYAACIG----ANLGHCAPASSAGSLAAGPLLLALALYPLSVLF--
p        PHVMAFAACIEPYTDCNLGNGASSQQFPTKCSWSALIAFVIILLTEKIMS
         *****:****     .***: *.:..  : .: . *:*:.:  *:  :
```
