# Supplementary material for: Immunolocalization of Some Epidermal Proteins and Glycoproteins in the Growing Skin of the Australian Lungfish (Neoceratodus forsteri)
Source: J Dev Biol. 2023 Aug 14;11(3):35. doi: 10.3390/jdb11030035 (PMC10443291; doi:10.3390/jdb11030035)
Supplement: Supplementary file 1 [file jdb-11-00035-s001.zip › Supplementary Figure Legends.pdf]

Supplementary Figure legends:

Supplementary Fig. 1 (S1). Clustal W comparison between the initial amino acid sequences (aa) of rat versus *P. annectens* nestin. This region is the more conserved between the two species. Stars represent identities (same amino acid), colons indicate substitutive but conservative amino acid replacements (amino acids with similar chemical-physical characteristics such as 3D-shape, size and solubility), dots indicate semi-conserved amino acid substitutions (amino acids with similar size but different polarity).

Supplementary Fig. 2 (S2). CLUSTAL-W comparison between human (h, AN AAC98395.1) and *Protopterus annectens* (p, AN XP_043910108.1) elastin, showing numerous common epitopes. . Stars represent identities (same amino acid), colons indicate substitutive but conservative amino acid replacements (amino acids with similar chemical-physical characteristics such as 3D-shape, size and solubility), dots indicate semi-conserved amino acid substitutions (amino acids with similar size but different polarity).

Supplementary Fig. 3 (S3). two examples of Clustal-w selected proteins form the bull (*Bos taurus*, b, the original antigen of the antibodies here utilized), and the amino acid sequences (n) identified in the genome of *Protopterus annectens* (p). **A**, is for osteonectin in *Bos taurus* (b) compared to a *P. annectens* osteonectin (p). **B**, is for human alkaline phosphatase (h) compared to that detected in *P. annectens* (p). Stars represent identities (same amino acid), colons indicate substitutive but conservative amino acid replacements (amino acids with similar chemical-physical characteristics such as 3D-shape, size and solubility), dots indicate semi-conserved amino acid substitutions (amino acids with similar size but different polarity).
